# 2-Amino-6-nitro-1*H*-benzoimidazol-3-ium chloride

**DOI:** 10.1107/S1600536809027342

**Published:** 2009-07-18

**Authors:** You-Sheng Chen, Kun Zhang, Su-Qing Zhao

**Affiliations:** a Faculty of Chemical Engineering and Light Industry, Guangdong University of Technology, Waihuan Xi Road No. 100, Guangzhou Higher Education Mega Center, Panyu District, Guangzhou, Guangzhou 510006, People’s Republic of China

## Abstract

In the cation of the title compound, C_7_H_7_N_4_O_2_^+^·Cl^−^, the benzimidazole ring system is planar with a maximum deviation of −0.019 (3) Å. In the crystal structure, C—H⋯Cl, N—H⋯Cl, and N—H⋯Cl inter­actions link the mol­ecules into a two-dimensional network. π–π contacts between benzimidazole rings [centroid–centroid distances = 3.928 (1) and 3.587 (1) Å] may further stabilize the structure.

## Related literature

For bond-length data, see: Allen *et al.* (1987 ▶).
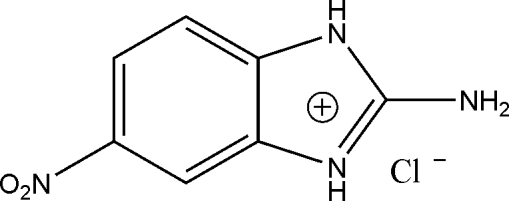


## Experimental

### 

#### Crystal data

C_7_H_7_N_4_O_2_^+^·Cl^−^
*M* *_r_* = 214.62
Monoclinic, 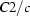

*a* = 13.969 (3) Å
*b* = 7.8064 (19) Å
*c* = 16.490 (4) Å
β = 91.303 (3)°
*V* = 1797.7 (7) Å^3^
*Z* = 8
Mo *K*α radiation
μ = 0.40 mm^−1^
*T* = 291 K
0.12 × 0.12 × 0.10 mm

#### Data collection

Enraf–Nonius CAD-4 diffractometer
Absorption correction: ψ scan (North *et al.*, 1968 ▶) *T*_min_ = 0.953, *T*_max_ = 0.961
4345 measured reflections
1580 independent reflections
1242 reflections with *I* > 2σ(*I*)
*R*_int_ = 0.041
3 standard reflections frequency: 120 min intensity decay: none

#### Refinement

*R*[*F* ^2^ > 2σ(*F* ^2^)] = 0.035
*wR*(*F* ^2^) = 0.096
*S* = 1.02
1580 reflections
127 parameters
H-atom parameters constrained
Δρ_max_ = 0.18 e Å^−3^
Δρ_min_ = −0.20 e Å^−3^

### 

Data collection: *CAD-4 Software* (Enraf–Nonius, 1989 ▶); cell refinement: *CAD-4 Software*; data reduction: *XCAD4* (Harms & Wocadlo, 1995 ▶); program(s) used to solve structure: *SHELXS97* (Sheldrick, 2008 ▶); program(s) used to refine structure: *SHELXL97* (Sheldrick, 2008 ▶); molecular graphics: *ORTEP-3 for Windows* (Farrugia, 1997 ▶) and *PLATON* (Spek, 2009 ▶); software used to prepare material for publication: *SHELXL97* and *PLATON*.

## Supplementary Material

Crystal structure: contains datablocks global, I. DOI: 10.1107/S1600536809027342/hk2728sup1.cif

Structure factors: contains datablocks I. DOI: 10.1107/S1600536809027342/hk2728Isup2.hkl

Additional supplementary materials:  crystallographic information; 3D view; checkCIF report

## Figures and Tables

**Table 1:** Hydrogen-bond geometry (Å, °)

*D*—H⋯*A*	*D*—H	H⋯*A*	*D*⋯*A*	*D*—H⋯*A*
C7—H7⋯Cl1	0.93	2.75	3.436 (2)	132
N1—H1*A*⋯Cl1	0.86	2.61	3.2830 (19)	135
N1—H1*A*⋯Cl1^i^	0.86	2.76	3.4102 (19)	134
N3—H3*A*⋯Cl1^ii^	0.86	2.55	3.269 (2)	142
N2—H2*A*⋯Cl1^ii^	0.86	2.31	3.0601 (19)	145

Symmetry codes: (i) 
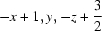
; (ii) 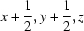.

**Fig. 1.**
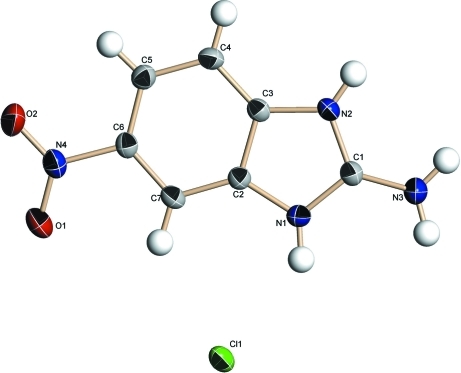
The molecular structure of the title molecule, with the atom-numbering scheme. Displacement ellipsoids are drawn at the 50% probability level.

**Fig. 2.**
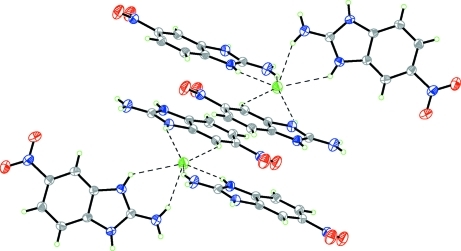
A partial packing diagram of the title compound. Hydrogen bonds are shown as dashed lines.

## supplementary crystallographic information

### Comment

Some derivatives of 2-aminobenzimidazolium are important chemical materials. We report herein the crystal structure of the title compound.

In the molecule of the title compound, (Fig. 1), the bond lengths (Allen *et al.*,  1987) and angles are within normal ranges. Rings A (N1/N2/C1-C3) and B (C2-C7) are, of course, planar and they are oriented at a dihedral angle of 1.70 (3)°. The benzimidazole ring system is planar with a maximum deviation of -0.019 (3) Å for atom C5. Atoms O1, N3 and N4 are 0.064 (3), -0.044 (3) and -0.094 (3) Å away from the plane of the benzimidazole ring system, respectively.

In the crystal structure, intramolecular C-H···Cl and N-H···Cl and intermolecular N-H···Cl interactions (Table 1) link the molecules into a two dimensional network (Fig. 2), in which they may be effective in the stabilization of the structure. The π–π contacts between the benzimidazole rings, Cg1—Cg2^i^ and Cg2—Cg2^ii^ [symmetry codes: (i) 1/2 - x, 3/2 - y, -z, (ii) -x, 1 - y, -z, where Cg1 and Cg2 are centroids of the rings A (N1/N2/C1-C3) and B (C2-C7), respectively] may further stabilize the structure, with centroid-centroid distances of 3.928 (1) and 3.587 (1) Å, respectively.

### Experimental

For the preparation of the title compound, a suspension of 4-nitro-*o*-phenylenediamine (1.4 g, 9.1 mmol) in a solution of BrCN (0.97 g, 9.2 mmol) in water (30 ml) was refluxed for 7 h, and then cooled and neutralized with NH_4_OH (25%) to pH = 11. The formed precipitate was filtered, washed with water and dried to give the title compound, as a yellow solid (yield; 1.5 g, 92%). Crystals suitable for X-ray analysis were obtained after 3 d by slow evaporation of the mother liquid at room temperature.

### Refinement

H atoms were positioned geometrically, with N-H = 0.86 Å (for NH and NH_2_) and C-H = 0.93 Å for aromatic H, respectively, and constrained to ride on their parent atoms, with U_iso_(H) = 1.2U_eq_(C,N).

### Crystal data

**Table:**

C_7_H_7_N_4_O_2_^+^·Cl^−^	*F*(000) = 880
*M**_r_* = 214.62	*D*_x_ = 1.586 Mg m^−^^3^
Monoclinic, *C*2/*c*	Mo *K*α radiation, λ = 0.71073 Å
Hall symbol: -C 2yc	Cell parameters from 25 reflections
*a* = 13.969 (3) Å	θ = 2.1–25.3°
*b* = 7.8064 (19) Å	µ = 0.40 mm^−^^1^
*c* = 16.490 (4) Å	*T* = 291 K
β = 91.303 (3)°	Block, yellow
*V* = 1797.7 (7) Å^3^	0.12 × 0.12 × 0.10 mm
*Z* = 8	

### Data collection

**Table:**

Enraf–Nonius CAD-4 diffractometer	1242 reflections with *I* > 2σ(*I*)
Radiation source: fine-focus sealed tube	*R*_int_ = 0.041
graphite	θ_max_ = 25.0°, θ_min_ = 2.5°
ω/2θ scans	*h* = −16→16
Absorption correction: ψ scan (North *et al.*, 1968)	*k* = −9→9
*T*_min_ = 0.953, *T*_max_ = 0.961	*l* = −19→13
4345 measured reflections	3 standard reflections every 120 min
1580 independent reflections	intensity decay: none

### Refinement

**Table:**

Refinement on *F*^2^	Primary atom site location: structure-invariant direct methods
Least-squares matrix: full	Secondary atom site location: difference Fourier map
*R*[*F*^2^ > 2σ(*F*^2^)] = 0.035	Hydrogen site location: inferred from neighbouring sites
*wR*(*F*^2^) = 0.096	H-atom parameters constrained
*S* = 1.02	*w* = 1/[σ^2^(*F*_o_^2^) + (0.0429*P*)^2^ + 0.969*P*] where *P* = (*F*_o_^2^ + 2*F*_c_^2^)/3
1580 reflections	(Δ/σ)_max_ < 0.001
127 parameters	Δρ_max_ = 0.18 e Å^−^^3^
0 restraints	Δρ_min_ = −0.19 e Å^−^^3^

### Special details

**Table:**

Geometry. All e.s.d.'s (except the e.s.d. in the dihedral angle between two l.s. planes) are estimated using the full covariance matrix. The cell e.s.d.'s are taken into account individually in the estimation of e.s.d.'s in distances, angles and torsion angles; correlations between e.s.d.'s in cell parameters are only used when they are defined by crystal symmetry. An approximate (isotropic) treatment of cell e.s.d.'s is used for estimating e.s.d.'s involving l.s. planes.
Refinement. Refinement of *F*^2^ against ALL reflections. The weighted *R*-factor *wR* and goodness of fit *S* are based on *F*^2^, conventional *R*-factors *R* are based on *F*, with *F* set to zero for negative *F*^2^. The threshold expression of *F*^2^ > σ(*F*^2^) is used only for calculating *R*-factors(gt) *etc*. and is not relevant to the choice of reflections for refinement. *R*-factors based on *F*^2^ are statistically about twice as large as those based on *F*, and *R*- factors based on ALL data will be even larger.

### Fractional atomic coordinates and isotropic or equivalent isotropic displacement parameters (Å^2^)

**Table:**

	*x*	*y*	*z*	*U*_iso_*/*U*_eq_	
Cl1	0.40318 (4)	0.59647 (8)	0.67245 (4)	0.0535 (2)	
O1	0.38248 (13)	0.6546 (3)	0.39469 (12)	0.0718 (6)	
O2	0.42766 (13)	0.8483 (3)	0.31128 (11)	0.0743 (6)	
N1	0.61668 (12)	0.7489 (2)	0.63485 (10)	0.0424 (5)	
H1A	0.5835	0.6840	0.6656	0.051*	
N2	0.72946 (12)	0.9189 (2)	0.59344 (10)	0.0403 (4)	
H2A	0.7805	0.9804	0.5934	0.048*	
N3	0.73958 (14)	0.8248 (3)	0.72889 (12)	0.0552 (6)	
H3A	0.7916	0.8812	0.7380	0.066*	
H3B	0.7150	0.7649	0.7669	0.066*	
N4	0.43847 (13)	0.7660 (3)	0.37360 (12)	0.0491 (5)	
C1	0.69763 (15)	0.8301 (3)	0.65691 (13)	0.0392 (5)	
C2	0.59515 (14)	0.7865 (3)	0.55416 (12)	0.0363 (5)	
C3	0.66716 (14)	0.8960 (3)	0.52782 (13)	0.0361 (5)	
C4	0.66616 (16)	0.9615 (3)	0.44987 (13)	0.0420 (5)	
H4	0.7147	1.0332	0.4323	0.050*	
C5	0.59021 (16)	0.9163 (3)	0.39925 (13)	0.0432 (5)	
H5	0.5859	0.9590	0.3466	0.052*	
C6	0.52047 (15)	0.8067 (3)	0.42752 (13)	0.0392 (5)	
C7	0.52051 (15)	0.7385 (3)	0.50434 (13)	0.0401 (5)	
H7	0.4728	0.6644	0.5213	0.048*	

### Atomic displacement parameters (Å^2^)

**Table:**

	*U*^11^	*U*^22^	*U*^33^	*U*^12^	*U*^13^	*U*^23^
Cl1	0.0422 (4)	0.0708 (4)	0.0475 (4)	−0.0144 (3)	0.0034 (3)	−0.0083 (3)
O1	0.0580 (12)	0.0805 (13)	0.0762 (13)	−0.0252 (10)	−0.0151 (10)	0.0019 (11)
O2	0.0612 (12)	0.1070 (16)	0.0539 (12)	−0.0053 (11)	−0.0168 (9)	0.0130 (11)
N1	0.0447 (11)	0.0431 (11)	0.0393 (11)	−0.0091 (9)	0.0011 (8)	0.0035 (8)
N2	0.0328 (10)	0.0461 (11)	0.0419 (10)	−0.0076 (8)	−0.0012 (8)	0.0000 (8)
N3	0.0523 (13)	0.0663 (14)	0.0466 (12)	−0.0117 (10)	−0.0096 (10)	0.0075 (10)
N4	0.0387 (11)	0.0593 (13)	0.0491 (12)	0.0016 (10)	−0.0039 (9)	−0.0090 (10)
C1	0.0351 (11)	0.0413 (12)	0.0411 (13)	0.0005 (9)	−0.0013 (9)	−0.0016 (10)
C2	0.0357 (11)	0.0366 (11)	0.0367 (12)	0.0003 (9)	0.0019 (9)	−0.0012 (9)
C3	0.0311 (11)	0.0368 (11)	0.0405 (12)	0.0005 (9)	0.0022 (9)	−0.0035 (9)
C4	0.0372 (12)	0.0445 (13)	0.0446 (13)	−0.0050 (10)	0.0064 (10)	0.0019 (10)
C5	0.0430 (13)	0.0479 (13)	0.0387 (12)	0.0007 (10)	0.0026 (10)	0.0006 (10)
C6	0.0364 (12)	0.0420 (12)	0.0391 (12)	0.0023 (9)	−0.0021 (9)	−0.0063 (10)
C7	0.0362 (12)	0.0393 (12)	0.0448 (13)	−0.0059 (9)	0.0039 (10)	−0.0051 (10)

### Geometric parameters (Å, °)

**Table:**

N1—H1A	0.8600	C2—C7	1.365 (3)
N2—H2A	0.8600	C3—N2	1.385 (3)
N3—H3A	0.8600	C3—C4	1.383 (3)
N3—H3B	0.8600	C4—C5	1.381 (3)
N4—O1	1.225 (3)	C4—H4	0.9300
N4—O2	1.219 (2)	C5—C6	1.385 (3)
C1—N1	1.340 (3)	C5—H5	0.9300
C1—N2	1.340 (3)	C6—C7	1.374 (3)
C1—N3	1.312 (3)	C6—N4	1.469 (3)
C2—N1	1.389 (3)	C7—H7	0.9300
C2—C3	1.397 (3)		
			
C1—N1—C2	108.84 (17)	C7—C2—C3	121.8 (2)
C1—N1—H1A	125.6	N2—C3—C2	106.28 (18)
C2—N1—H1A	125.6	C4—C3—N2	132.2 (2)
C1—N2—C3	109.26 (17)	C4—C3—C2	121.6 (2)
C1—N2—H2A	125.4	C3—C4—H4	121.4
C3—N2—H2A	125.4	C5—C4—C3	117.3 (2)
C1—N3—H3A	120.0	C5—C4—H4	121.4
C1—N3—H3B	120.0	C4—C5—C6	119.4 (2)
H3A—N3—H3B	120.0	C4—C5—H5	120.3
O1—N4—C6	118.4 (2)	C6—C5—H5	120.3
O2—N4—O1	123.1 (2)	C5—C6—N4	118.3 (2)
O2—N4—C6	118.5 (2)	C7—C6—N4	117.27 (19)
N2—C1—N1	109.02 (18)	C7—C6—C5	124.3 (2)
N3—C1—N1	126.0 (2)	C2—C7—C6	115.6 (2)
N3—C1—N2	125.0 (2)	C2—C7—H7	122.2
N1—C2—C3	106.60 (18)	C6—C7—H7	122.2
C7—C2—N1	131.6 (2)		

### Hydrogen-bond geometry (Å, °)

**Table:**

*D*—H···*A*	*D*—H	H···*A*	*D*···*A*	*D*—H···*A*
C7—H7···Cl1	0.93	2.75	3.436 (2)	132
N1—H1A···Cl1	0.86	2.61	3.2830 (19)	135
N1—H1A···Cl1^i^	0.86	2.76	3.4102 (19)	134
N3—H3A···Cl1^ii^	0.86	2.55	3.269 (2)	142
N2—H2A···Cl1^ii^	0.86	2.31	3.0601 (19)	145

Symmetry codes: (i) −*x*+1, *y*, −*z*+3/2; (ii) *x*+1/2, *y*+1/2, *z*.

## References

[bb1] Allen, F. H., Kennard, O., Watson, D. G., Brammer, L., Orpen, A. G. & Taylor, R. (1987). *J. Chem. Soc. Perkin Trans. 2*, pp. S1–19.

[bb2] Enraf–Nonius (1989). *CAD-4 Software* Enraf–Nonius, Delft, The Netherlands.

[bb3] Farrugia, L. J. (1997). *J. Appl. Cryst.* **30**, 565.

[bb4] Harms, K. & Wocadlo, S. (1995). *XCAD4* University of Marburg, Germany.

[bb5] North, A. C. T., Phillips, D. C. & Mathews, F. S. (1968). *Acta Cryst.* A**24**, 351–359.

[bb6] Sheldrick, G. M. (2008). *Acta Cryst.* A**64**, 112–122. 10.1107/S0108767307043930 18156677

[bb7] Spek, A. L. (2009). *Acta Cryst.* D**65**, 148–155. 10.1107/S090744490804362X PMC2631630 19171970

